# When Nurses Worry: A Concept Analysis of Intuition in Clinical Deterioration

**DOI:** 10.1111/jan.16956

**Published:** 2025-04-10

**Authors:** Amy‐Louise Byrne, Deb Massey, Tracy Flenady, Justine Connor, Wei Ling Chua, Danielle Le Lagadec

**Affiliations:** ^1^ School of Nursing, Midwifery and Social Sciences CQUniversity Rockhampton Queensland Australia; ^2^ Edith Cowen University Joondalup Western Australia Australia; ^3^ Alice Lee Centre for Nursing Studies, Yong Loo Lin School of Medicine National University of Singapore Singapore

**Keywords:** clinical deterioration, concept analysis, escalation, nurse worry

## Abstract

**Background:**

Nurse worry is a criterion often included in early warning systems tools and used to escalate care when other clinical markers do not indicate deterioration. What it means to worry, however, is not always clear.

**Aims:**

To generate a concept analysis of nurse worry in relation to clinical deterioration.

**Design:**

Rodgers's evolutionary method was used.

**Method:**

A review was first conducted in April 2024, searching the Cumulative Index for Nursing and Allied Health Literature, Pubmed, EmCare, and Embase databases. A total of 22 articles were subjected to analysis of the antecedents, attributes, and consequences of nurse worry in the context of clinical deterioration. The processes of nurse worry were subsequently mapped and conceptualised, leading to a descriptive statement of nurse worry.

**Results:**

Worry captures a nurse's sense of knowing the patient and is embodied via assessing, sensing, recognising, and processing information, cues, and patterns.

**Conclusion:**

Nurse worry is a complex process, impacted by external and internal factors. Implications for the profession or patient care: Assured practice, driven by validation of a nurse's worry, leads to proactive care of the deteriorating patient, whereas apprehensive practice, driven by fear and trepidation, leads to reactive care of the deteriorating patient.

**Impact:**

Nurse worry is a criterion often included in early warning systems tools and used to escalate care when other clinical markers do not indicate clinical deterioration. What it means to worry, however, is not always clear. From the concept analysis, a descriptive statement of nurse worry emerged. Worry captures a nurse's sense of knowing the patient and is embodied via assessing, sensing, recognising, and processing information, cues, and patterns.

**Implications for the Profession or Patient Care:**

This research has implications for nurses, policymakers, and organisations, as nurse worry is an important element in detecting clinical deterioration. Nurse worry must be organisationally supported.

**Reporting Methods:**

PRISMA Extension for Scoping Reviews (PRISMA‐SCR).

**Patient or Public Contribution:**

No Patient or Public Contribution.

**Summary**

- What does this paper contribute to the wider global clinical community?
  ○ Provides a descriptive statement of nurse worry.
  ○ Provides a conceptualisation of the nurse worry process.
  ○ Identifies key drivers of nurse worry which support safe, quality practice.

## 1 Introduction

For more than two decades, the appropriate detection and management of clinical deterioration has been of key importance for quality, safe care provision within health services (DeVita et al. 2017). Early recognition and response to clinical deterioration are crucial in preventing serious adverse events such as unplanned intensive care unit (ICU) admissions, cardiac arrest, and even death (Jones et al. 2011). This has led to the development of physiological track‐and‐trigger systems (PTTSs) in hospitals to support nurses in the early recognition of clinical deterioration and guide the escalation of care decisions and clinical actions to prevent further deterioration (Gao et al. 2007).

In general, the ‘tracking’ component of PTTSs is based on the patient's physiological parameters, such as respiratory rate, oxygen saturation and requirement, heart rate, blood pressure, temperature, and sometimes includes pain and level of consciousness (Gao et al. 2007). Depending on the needs and available resources of individual healthcare settings, PTTS can be in the form of (1) an aggregate weighted scoring system where a cumulative score is generated based on the severity of deviations in multiple clinical parameters, e.g., the United Kingdom's National Early Warning Score 2 (NEWS2), (2) a single parameter system where a breach of any single clinical parameter would trigger a clinical response, e.g., the Australian Between the Flags (BTF), or (3) a combination system where a single parameter system is used in combination with an aggregate weighted scoring system, e.g., Queensland Adult Deterioration Detection System (Q‐ADDS) (Le Lagadec and Dwyer 2017).

These objective and quantifiable means of identifying deterioration have faced criticism for devaluing the importance of nurses' intuitive senses and subjective judgement in recognising early signs of deterioration (Chua et al. 2020; Osborne et al. 2015). Several studies have demonstrated that nurses' worry, often grounded in their clinical experience, knowledge, and observations of subtle changes in a person's behaviour, appearance, or overall condition, can frequently precede measurable changes in vital signs (Chua et al. 2019; Cioffi 2000b; Odell et al. 2009). This underscores the crucial role of nurses' worry in the early recognition of clinical deterioration, making it a valuable complement to objective‐based PTTS (Douw et al. 2015).

Acknowledging the value of nurses' worry in detecting early signs of clinical deterioration, some PTTS have included a ‘worried’ criterion, which allows nurses to escalate care based on their intuition that a patient is deteriorating even if vital signs remain within normal ranges (Douw et al. 2017; Romero‐Brufau et al. 2019). Although nurses' worry in the context of clinical deterioration has appeared in the nursing literature for decades, the worry concept remains poorly defined, poorly understood, and poorly conceptualised, making it difficult to capture its impact on outcomes.

## 2 Background

PTTSs were introduced to facilitate early detection of clinical deterioration and prompt escalation of treatment (Flenady et al. 2020). These systems typically include detection tools that guide nursing actions based on the patient's clinical parameters. Many PTTSs employ colour coding patient observation charts and scoring systems to indicate the severity of vital signs derangement. For instance, a score of three (3) on Q‐ADDS may prompt a nurse to repeat vital signs within an hour, while a score of eight (8) would necessitate an emergency call (Preece et al. 2010). However, reliance on these numerical scores or colour codes risks overlooking nurses' heuristic and experiential knowledge, which often serves as an early indicator of patient decline (Douw et al. 2018). PTTSs that are based on objective physiological data may fail to capture subtle patterns or an intuitive sense of worry that nurses often develop through their knowledge of the patient (Romero‐Brufau et al. 2019).

Nurses are well‐placed to identify subtle clinical changes in patients before objective, measurable signs of clinical compromise are evident (Massey et al. 2016). However, the escalation of a person's condition can be hindered by nurses not raising their concerns (Lee et al. 2021) and there is increasing awareness of the factors inhibiting nurses from escalating care (Massey et al. 2014; Shearer et al. 2012). Burrell et al. (2020) reported that empowering nurses and validating their concerns are effective mechanisms for improving early recognition and timely intervention for at‐risk patients. This was the impetus of a novel rapid response team (RRT), which implemented a nurse practitioner (NP)‐led responder instead of the typical physician‐led RRT (Burrell et al. 2020). NP‐led RRT improved care escalation driven by nurse concern, fostering better communication and trust between bedside nurses and NP‐led RRT (Burrell et al. 2020). Importantly, it also addressed a major barrier, nurses' fear of speaking up, leading to more proactive RRT evaluations before major clinical deterioration occurred (Burrell et al. 2020).

Creating pathways for nurses to raise their concerns is paramount to patient safety (Lee et al. 2021). A recent qualitative study by Burke and Conway (2023) found that nurses use their own process when deciding to escalate care, finding a balance between using their clinical judgement and PTTS. While PTTSs are valued for identifying deteriorating patients and lending credibility to concerns when speaking with multidisciplinary colleagues, nurses often find it somewhat restrictive, particularly when relying on their intuition and clinical judgement in recognising clinical deterioration (Burke and Conway 2023; Massey et al. 2024). This is important because nurses are with the patients in a far greater capacity than other healthcare clinicians, allowing them to pick up subtle changes over time (Agostinho et al. 2023).

A retrospective review of RRT activations at a single facility revealed that bedside nurses were typically the ones initiating the activations, with nearly 25% of the activations driven by nurse worry or concerns rather than objective triggers (Bunch et al. 2019). This suggests that nurse worry or concern contributes significantly to their decision‐making. ‘Worried’ is sufficiently important to have instigated its inclusion as an indicator of clinical decline when designing PTTS (Bunch et al. 2019).

Despite the recognised importance of nurse worry, its application in clinical practice remains inconsistent, largely due to the absence of a clear definition. Shearer et al. (2012) found that local workplace culture heavily influences nurses' responses to clinical deterioration, and failure to adhere to established RRT activation protocols can result in failure to rescue. Many nurses in their study did not activate the RRT despite being concerned about the patient's condition and knowing the criteria for RRT activation were met (Shearer et al. 2012).

Acknowledging this gap, we aimed to conceptually analyse the construct of ‘nurse worry’, exploring its definition, application, and role in clinical decision‐making.

## 3 The Study

The research question was driven by the existing limitations in understanding nurse worry and how it is operationalised in the clinical setting. As such, the research question was:

‘How does nurse worry function as an early indicator of clinical deterioration in healthcare settings?’

### 3.1 Aims and Objectives

This study aimed to undertake a concept analysis of nurse worry in relation to clinical deterioration. Using Rodgers's evolutionary method (Rodgers 2000), the objectives were as follows:

- Collate the surrogate terms, attributes, antecedents, and consequences of ‘nurse worry’ in the context of clinical deterioration.
- Analyse the findings in the context of clinical deterioration and related nursing processes.
- Develop an operational definition of the term within the context.

### 3.2 Design

Rodger's evolutionary concept analysis was selected for this review, given its systematic method and applicability in exploring concepts with fluid and dynamic meanings (Rodgers and Knafl 2000). A strength of Rodgers' approach is that it uses inductive reasoning, allowing for how concepts evolve over time and in relation to context. In comparison to other, more structured methods such as Walker and Avant (2005), Rodgers' method is flexible and non‐linear in its design, allowing for simultaneous activities to be undertaken during analysis (Gunawan et al. 2023). This inductive method of data analysis supports rich investigation and development of concepts, providing greater applicability for those in the contemporary health sphere (Rodgers 2000). This approach has been used to develop and guide other important nursing concepts, such as clinical judgement (Connor et al. 2022). Given the topic (nurse worry and clinical deterioration), it was determined that a flexible and more fluid framework would be advantageous for this analysis.

As defined by Rodgers (2000), six phases were used to explore and conceptualise nurse worry (see Table 1). It is important to note that these phases are not necessarily linear but iterative in nature.

**TABLE 1:** Rodgers six phase concept analysis framework (Rodgers 2000).

Phase 1	Identify surrogate terminology and relevant uses of the concept
Phase 2	Identify and select an appropriate setting (time period) and sample for data collection
Phase 3	Collect data—attributes, antecedents, and consequences on a contextual basis of the concept
Phase 4	Analyse the data regarding the above characteristics
Phase 5	Identify an exemplar of the concept if appropriate
Phase 6	Identify implications for further development of the concept

### 3.3 Surrogate and Related Terms

The term ‘nurse worry’ is used in the clinical area, often related to a patient's clinical condition and deterioration. Surrogate terms include concern, indicating a nurse's state of being, in relation to having identified clinical parameters or other issues that they believe may impact the patient's health. Importantly, concern can be present where no other data, such as vital signs or test results, indicate deterioration (Douw et al. 2015). This leads to another group of surrogate terms associated with intuition.

Intuition is defined as the ability to instinctually synthesise information without conscious reasoning (Chilcote 2017). Nurses use their experiences and knowledge to develop intuition, allowing them to recognise the potential for deterioration and act on this before clinical parameters change (Odell et al. 2009). Research by Melin‐Johansson et al. (2017) found that nurse intuition included assertiveness and experiences, and when applying intuition, nurses drew on the connections they made with the patient, their mental and physical responses, and the personal qualities of the nurse. In terms of the nursing process, support and guidance are needed to validate decisions (Melin‐Johansson et al. 2017). Alternative terms include ‘gut feeling’ and having a sixth sense, which indicates ways of knowing that are embedded into professional instinct nurses develop over time. Intuition allows nurses to understand that something is wrong, and act on this feeling. It is part of a nurse's clinical judgement.

Clinical judgement is also used in the context of nurse worry. Connor et al., (2022, 3336) define clinical judgement as a ‘reflective and reasoning process that draws upon all available data, is informed by an extensive knowledge base and results in the formation of a clinical conclusion’. Indeed, clinical judgement is used in the context of deterioration but is positioned parallel to the concept of worry, which appears to drive the patient assessment process.

For the current study, Jones' et al. (2013, 1033) definition of deterioration was adopted: ‘A deteriorating patient is one who moves from one clinical state to a worse clinical state which increases their individual risk of morbidity, including organ dysfunction, protracted hospital stay, disability, or death.’

### 3.4 Data Collection

In consultation with a CQUniversity librarian, the search strategy was developed, and the following databases were searched: Cumulative Index for Nursing and Allied Health Literature (CINAHL), Pubmed, EmCare, and Embase databases. Based on the PICo (population, intervention, and context) framework, the surrogate terms and related Medical Subject Headings (MeSH) were used to develop the search strategy as shown in Table 2. The Boolean Operators AND and OR were used to link the search terms (see Table 2). The search string used in Pubmed is provided as an example: ((Nursing Staff) OR (registered general nurse) OR (licensed practical nurse) OR (staff nurse) OR (Registered nurses)) AND ((fifth vital sign) OR (something is not right) OR (intuitive knowledge) OR (Hunch) OR (something is off) OR (professional instinct) OR (sixth sense) OR (gut feeling) OR (“ways of knowing”) OR (clinical worry) OR (intuition) OR (Concern*) OR (Worried) OR (Worry)) AND ((acute deterioration) OR (detection of clinical deterioration) OR (patient deterioration)). The search was conducted by (DLL) on 30th April 2024, and the results were downloaded to EndNote.

**TABLE 2:** Search strategy.

Domain 1 (P)	Domain 2 (I)	Domain 3 (Co)
((((Nursing Staff) OR (registered general nurse)) OR (licensed practical nurse)) OR (staff nurse)) OR (Registered nurses)	(((((((((((((((fifth vital sign) OR (something is not right)) OR (intuitive knowledge)) OR (Hunch)) OR (something is off)) OR (professional instinct)) OR (sixth sense)) OR (witch sense)) OR (gut feeling)) OR (“ways of knowing”)) OR (clinical worry))) OR (intuition)) OR (Concern*)) OR (Worried)) OR (Worry)	((acute deterioration) OR (detection of clinical deterioration)) OR (patient deterioration)

**FIGURE 1:**
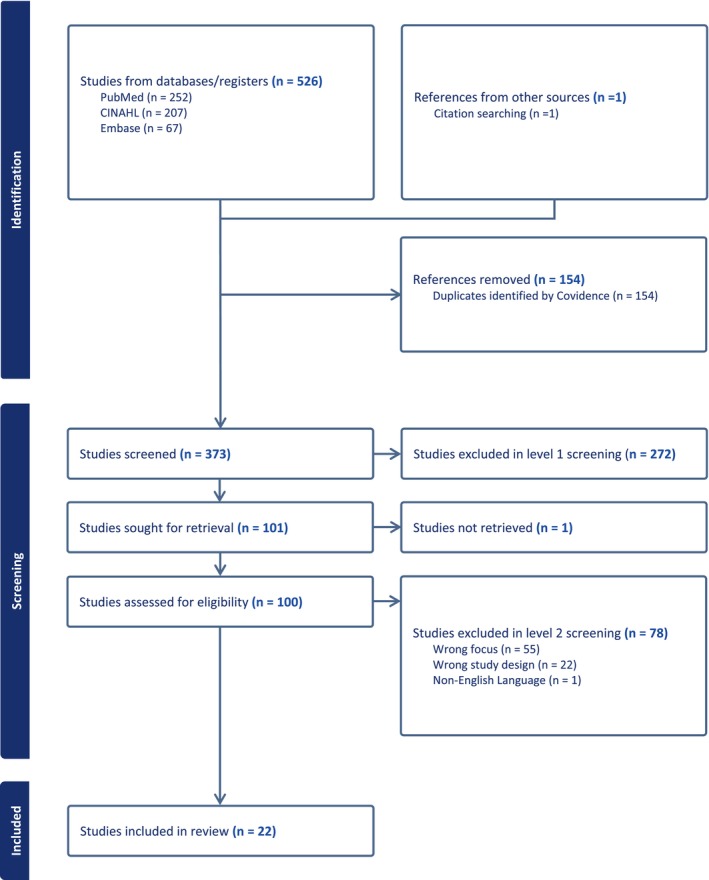
PRISMA flowchart (Covidence).

Full text, English language, peer‐reviewed articles were eligible for inclusion, with no geographical or time restrictions. Articles were included if they related to the conceptual understanding of nurse worry (or synonyms) and if they related to a definition, antecedents (exists before or precedes), attributes (features and characteristics), or consequences (the results or effect of worry) of the concept. Articles that were not peer‐reviewed or were opinion pieces/editorials were categorised as ‘wrong study design’ and excluded. Articles that did not focus on the worried concept or were simply descriptive were also excluded (categorised as ‘wrong focus’ in Figure 1). Articles related to midwives, mental health, or medical professions other than nursing were excluded under the category of ‘wrong focus’. A hand search of the reference lists of included studies was also carried out. Article screening was conducted using Covidence software, allowing for independent blinded review and categorisation of each article. Covidence also allowed for conflicts to be resolved by consensus. The results are shown in the PRIMSA flowchart (Figure 1) (Data S1).

The database search resulted in 526 articles, and an additional article was included following the hand search. After removal of duplicates, 373 articles were eligible for screening. Using the inclusion and exclusion criteria, the initial level 1 screening of titles and abstracts was conducted by (ALB and JC). A total of 100 articles were included for the full‐text, level 2 screening.

The second and final round of screening included an assessment of the full text articles. The articles were divided between four members of the research team, with every article being independently reviewed by two members of the team (ALB, JC, DLL, and WLC). Conflicting reviews were decided by team consensus. A total of 22 articles were included for final conceptual analysis.

### 3.5 Rigour

A concept analysis may choose a cross section of materials from a variety of locations, sources, and authors, as the goal is to better understand how the concept is understood and enacted (Rodgers 2000). While a formal quality appraisal was not required for the concept analysis, the design was careful to follow a robust methodology for searching and selecting articles. Additional measures, such as clear inclusion and exclusion criteria and blinded review by multiple authors, ensured rigour in selection.

### 3.6 Data Extraction and Analysis

**FIGURE 2:**
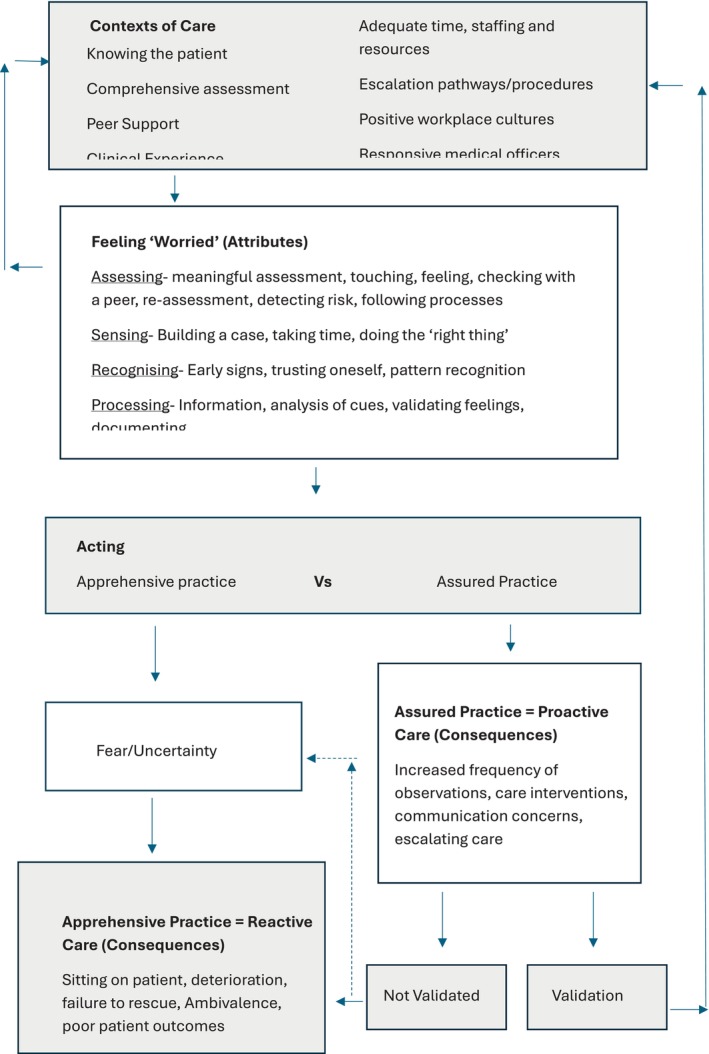
Conceptual model of nurse worry.

All selected articles were reviewed and ordered/extracted in relation to their attributes, antecedents, and consequences by two members of the research team (DM and TF). The data was extracted onto a purpose‐built extraction tool based on Rodgers' framework (Appendix A) and independently verified by another member of the research team (AB). The results were analysed through an iterative process in relation to the greater clinical context to determine a descriptive statement by two members of the team (AB and JC). Initial codes in relation to the attributes, antecedents, and consequences were generated and then considered regarding their relationship to one another (e.g., their order, logical progression, and outcomes). The analysis was validated by a separate research team member (DM). A summary of the included articles and the attributes, antecedents, and consequences is provided in Appendix A. Conceptual mapping allowed the analysis to take on greater meaning for nurse worry in the context of clinical deterioration, as depicted in Figure 2 below.

## 4 Findings

Nurse worry is a complex process. It is intuitive *and* deductive and closely linked to quality care and efficacy outcomes in the context of deterioration. Figure 2 demonstrates a conceptual model of the antecedents, attributes, and consequences of worry, in the context of deterioration.

### 4.1 Surrogate Terms

Rodgers' framework begins by identifying surrogate terms. According to Rodgers (2000), linguistic bearings impact the understanding of concepts within a complex manifestation of terms. Surrogate terms were identified as part of the search strategy (see Table 2) and were recaptured through the analysis process, as per the findings presented in Appendix A. The surrogate terms included an intuitive stance, e.g., intuition, sense, and knowing. For nurses, this captures knowledge that may not be easily measured or is more difficult to define and describe.

**FIGURE 3:**
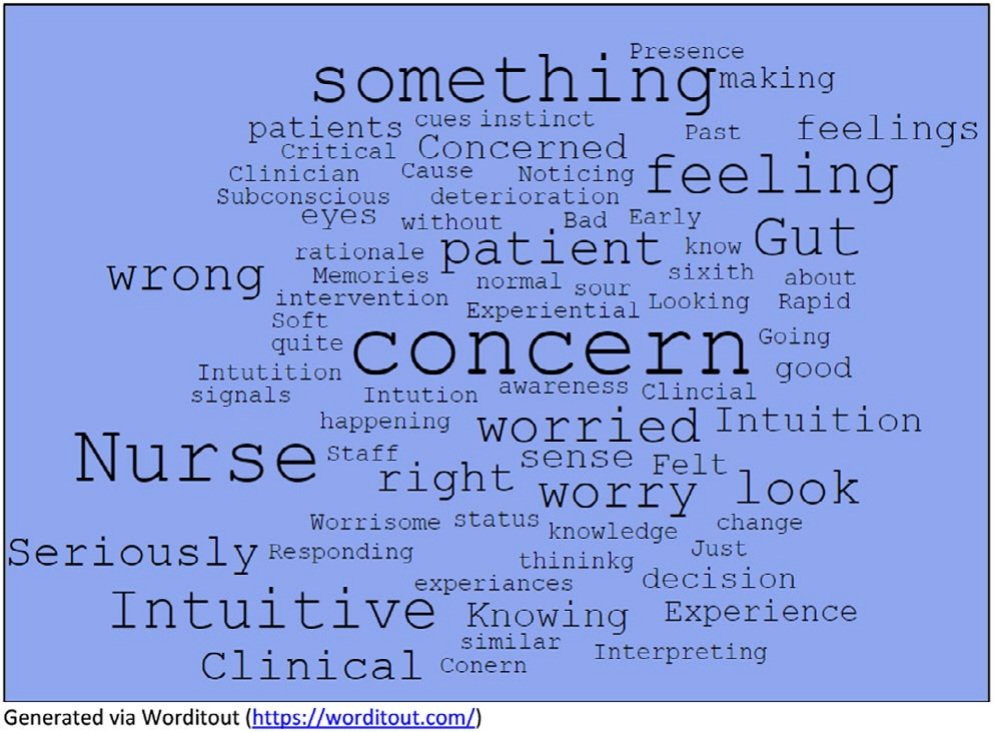
Worry surrogate terms. Generated via Worditout (https://worditout.com/).

Additionally, surrogate terms could be more deductive, such as judgement, clinical concern, and not normal. These terms denote the more tangible nursing actions of assessment, processing of data, and use of clinical tools and parameters. These surrogate terms are important, as the broader discourse around a term influences how it is understood and enacted. Figure 3 demonstrates an overview of these surrogate terms in the form of a word‐cloud. These terms and their meanings comprise the antecedents and attributes of worry and the resulting consequences.

### 4.2 Antecedents

The analysis of the antecedents from the included studies identified the need for supportive contexts of care (e.g., Ede et al. 2021) which can generate a sense of worry. Indeed, it is the context that drives and supports nurses' worry. These contexts were ordered into nursing support and organisational and team support.

#### 4.2.1 Nursing Support

‘Knowing’ the patient is fundamental to nurse's worry. Cioffi (2000a) alluded to a meaningful nurse–patient relationship, whereby understanding the patients' unique circumstances allows the nurse to gain richer insights into their condition and care trajectory. Knowing the patient is the baseline from which nurses work, assess and process information (Ede et al. 2021). Knowing stems from engagement with the patient and including them in their care assessment (Cioffi 2000b). Given the intimate nature of the care provided, knowing the patient is intrinsic to the nursing role (Dalton et al. 2018; Douw et al. 2015). Langkjaer et al. (2021) spoke of the nurse's clinical gaze, using all their senses, integrating the patient physical assessment, including the vital signs, with knowing the patient and relying on past experiences.

‘Knowing’ facilitates a comprehensive assessment of the patient. Chua et al. (2023) stated that assessment, in the context of worry, goes beyond numbers, where subtle signs of deterioration might be observed. Comprehensive assessment is often done in the context of foreseeing risk (Douw et al. 2016). Douw et al. (2018) described knowing without rationale, whereby deterioration might not yet be indicatable. However, a nurse's observations, beyond vital signs, elevated them to a state of heightened awareness.

Nurses leverage their clinical experience when considering the patient. Romero‐Brufau et al. (2019) reported that years of experience were associated with greater accuracy around worry. Past experiences support intuitive knowing (Odell et al. 2009). Experience contributes to the nursing gaze (Langkjaer et al. 2021) and nurses can use past experiences to reinforce or avoid specific actions, and to support decision‐making (Cioffi 2000a, 2000b; Dresser et al. 2023).

Nurses use peer support when considering deterioration (Peerboom et al. 2022). Peer support validates or authenticates worry (Dalton et al. 2018; Peerboom et al. 2022). Junior nurses require more peer support to facilitate decision‐making (Jensen et al. 2022), with some nurses justifying their worry through peer support (Raymond et al. 2019).

#### 4.2.2 Organisational Support

Organisational support is important in shaping the context of care, acting as an antecedent to nurses' worry. Nurses need time to form opinions and judgements during the deterioration event (Al‐Moteri et al. 2019; Minyaev et al. 2021) and require time to build confidence and gain experience (Douw et al. 2018; Jensen et al. 2022). Resources, such as available senior staff and appropriate skills mix within the team, are essential for fostering timely and accurate decision‐making (Dalton et al. 2018; Peerboom et al. 2022).

Our analysis identified a tension between nurses' judgement and PTTS escalation protocols (Minyaev et al. 2021), highlighting the need for clear, structured processes to guide patient monitoring, intervention and escalation of care. These processes are essential in supporting nurses' decision‐making (Douw et al. 2017; Ede et al. 2021; Odell et al. 2009). Organisational culture can either enable or hinder decision‐making and escalation of care (Dresser et al. 2023; Ede et al. 2021). A negative culture can instill a fear of incorrect decision‐making (Al‐Moteri et al. 2019), resulting in the undervaluation of nurses' clinical assessment (Cioffi 2000b).

The nurse–physician relationship is critical for decision‐making. Hesitancy or reluctance to communicate with physicians may delay escalation of care (Cioffi et al. 2009; Ede et al. 2021). This dynamic varies across cultural contexts and healthcare systems, influencing how escalation decisions are made and implemented. For instance, Chua et al. (2023) reported that in Asian healthcare settings, through familiarity with patients, nurses often detect deterioration before objective changes are evident. However, medical officers rely primarily on objective data, which can delay escalation. Clearly, there is a need to integrate both subjective and objective assessments while considering cultural and systemic factors.

### 4.3 Attributes

The attributes for nurse worry are assessing, sensing, recognising, and processing.

#### 4.3.1 Assessing

Assessment is both an antecedent and attribute of worry. As an attribute, assessment is primarily based on its meaningfulness. A meaningful assessment involves sensory and observational elements, such as feeling for a pulse, listening to breathing, observing changes in physical appearances (Langkjaer et al. 2021; Odell et al. 2009), and behavioural cues such as agitation. Objective clinical signs such as respiration rate, oxygenation, and blood pressure featured strongly in the literature (Odell et al. 2009; Peerboom et al. 2022; Cioffi 2000a; Cioffi et al. 2009, 2010; Douw et al. 2015).

We identified more subtle, subjective forms of assessment, ‘soft signs’ (Ede et al. 2021) like a usually boisterous patient being sombre (Dalton et al. 2018). Nurses often detect subtle cues through pattern recognition (Douw et al. 2016) and may respond by increasing the frequency of touch points with the patient (Dresser et al. 2023). Schnock et al. (2021) suggest actions such as frequent monitoring and documentation as indicators of nurse worried. As an attribute of worry, assessment is a complex mix of touching, feeling, sensing, and knowing; more than a soft skill, this is a careful marriage of clinical information and intuition (Jensen et al. 2022).

#### 4.3.2 Sensing

Sensing deterioration is an important process described as an awareness (Ede et al. 2021), a feeling (Al‐Moteri et al. 2019; Cioffi 2000a; Dalton et al. 2018; Jensen et al. 2022; Langkjaer et al. 2021) or a sixth sense (Cioffi 2000a). Sensing also taps into a nurse's sense of responsibility (Dresser et al. 2023), whereby nurses assume responsibility for care, detect deterioration and escalation, and worry is the first telltale of this responsibility.

#### 4.3.3 Recognising

Recognising clinical deterioration is deeply embedded within the worry paradigm (Dresser et al. 2023; Jensen et al. 2022). This includes a patient deviating from their expected care trajectory (Cioffi et al. 2010). Douw et al. (2018) described nurses anticipating deterioration and managing interventions and actions to address the deterioration. There is a sense of knowing something is about to happen (Cioffi 2000b; Dalton et al. 2018), triggered by something as nuanced as a look in the patient's eyes (Douw et al. 2018, 2015).

#### 4.3.4 Processing

Finally, worrying requires information processing, including subjective and objective data, cues, and information (Cioffi 2000b; Al‐Moteri et al. 2019). Non‐quantifiable evidence must be processed in the context of the individual (Cioffi et al. 2010). Nurses may reconcile this information by leveraging peer support (Douw et al. 2017; Dresser et al. 2023) or escalation protocols (Minyaev et al. 2021). Nurses reconcile this through internal processes (Odell et al. 2009), based on their experience, expertise, and judgement (Romero‐Brufau et al. 2019; Al‐Moteri et al. 2019; Douw et al. 2016).

While processing can be a deliberate action, nurses may also reconcile information unconsciously, making quick decisions (Douw et al. 2016). Processing information leads nurses to a decision‐making, using clinical judgement to progress care. These decisions can significantly impact care and lead to positive and negative consequences for patients and nurses.

### 4.4 Consequences

The consequences of nurse worry are subject to a critical junction. As per Figure 2, this is the point of action (acting) where nurses exercise their clinical judgement and make fundamental care decisions. A dichotomy emerged: assured practice with proactive care preventing a deterioration event versus apprehensive practice with reactive care responding to a deterioration event (see for example Al‐Moteri et al. 2019; Chua et al. 2023; and Douw et al. 2016, 2017). However, it is recognised that many variants exist in patient deterioration, and a dichotomous classification of decision‐making is overly simplistic.

#### 4.4.1 Assured Practice and Proactive Care

In assured practice, nurses enact their clinical judgement (Chua et al. 2023) through complex decision‐making (Cioffi 2000a). In practice, this involves transforming worry into action by presenting a compelling case for intervention (Al‐Moteri et al. 2019) and validating their findings and concerns (Dalton et al. 2018). This sometimes includes convincing physicians and the wider care teams that their worry is significant and requires intervention (Cioffi et al. 2010; Douw et al. 2017), reflecting a critical aspect of patient advocacy (Odell et al. 2009).

Assured practice leads to several responses including increasing patient monitoring (Douw et al. 2018) and instituting nurse‐initiated early interventions, such as starting fluids or repositioning a patient (Minyaev et al. 2021; Shiell et al. 2022). During a deterioration event, the assured practice stemming from nurse worry often leads to the escalation of care (Cioffi 2000a, 2000b) and the activation of the RRT (Dresser et al. 2023; Raymond et al. 2019), as depicted in Figure 2. In some cases, this results in a patient transfer to higher‐acuity settings (Romero‐Brufau et al. 2019). This is categorised as proactive care, a consequence of assured practice and nurse worry. When worry is supported at the nursing and organisational level and actions are taken proactively, nurses reported feeling validated in their concerns (Schnock et al. 2021). This validation reinforces their clinical judgement and legitimises their worry within the broader care team (Shiell et al. 2022).

#### 4.4.2 Apprehensive Practice

Contrariwise lies apprehensive and reactive care due to nurse worry. Nurses fear making the wrong decision (Al‐Moteri et al. 2019) and become anxious about expressing their feelings of worry (Chua et al. 2023). This anxiety impacts their clinical practice, with nurses expressing concerns that their opinions and clinical assessments are undervalued (Cioffi 2000a). Such experiences are frequently attributed to systemic issues, including poor workplace culture, exacerbating feelings of dread, fear and trepidation (Ede et al. 2021). This may affect nurses' ability and willingness to communicate concerns to physicians, potentially delaying care (Cioffi et al. 2009). A nurse's response can range from feelings of ambivalence about worry and the associated care (Chua et al. 2023) to waiting or ‘sitting’ on the patient (Cioffi 2000a), which may lead to failure to rescue (Al‐Moteri et al. 2019; Chua et al. 2023; Cioffi et al. 2009; Dalton et al. 2018; Douw et al. 2016, 2017). This is reactive care, a consequence of the apprehensive practice of nurse worry.

When worry is not supported at the nursing and organisational level, and actions are reactive, the consequences for patients, staff, and organisations can be dire. Apprehensive and reactive care is reinforced through fear, uncertainty, and trepidation.

### 4.5 Descriptive Statement

Rodgers' evolutionary analysis (Rodgers 2000) allows for the development of a descriptive statement. The concept analysis enables an in‐depth investigation of the meaning behind nurse worried. ‘Nurse worry’ can be defined through the context, attributes, and actions of care and impacts the quality of care provided to the patient. The analysis presents the contextual definition below. The bolded section offers a simplified meaning related to nursing practice.

The context of care must allow nurses the time, space, and support to escalate care concerns and facilitate a culture of safety, where worry is respected and acted upon by all staff. **Worry captures a nurse's sense of knowing the person/patient and is embodied via assessing, sensing, recognising, and processing information, cues, and patterns**. Assured practice, driven by validation of a nurse's worry, leads to proactive care of the deteriorating patient, whereas apprehensive practice, driven by fear and trepidation, leads to reactive care of the deteriorating patient.

## 5 Discussion

We sought to explore the concept of worry in the context of clinical deterioration. Using Rodgers' evolutionary method (Rodgers 2000), we have provided a nuanced understanding of worry in clinical practice, particularly how it influences nurses' perceptions and responses to patient deterioration. Nurse worry has been referred to as the fifth vital sign (Romero‐Brufau et al. 2019) emphasising its role and importance in recognising deterioration. Despite this, the worry concept remains poorly defined, poorly understood, and poorly conceptualised, making it difficult to capture its impact on patient outcomes, its importance to nursing practice, how the concept of nurse worry can be used to develop future policy initiatives, and how education providers can use the concept of nurse worry to inform healthcare curricula. The lack of a clearly articulated definition of nurse worry in the nursing curricula has failed to identify the role it plays in promoting patient safety. Therefore, nursing students have not been educated about the value and importance of the concept.

In developing this clear definition of worry, this concept analysis reveals the intricate relationship between nurse worry, nurses' knowledge, knowing the patient, and organisational support in relation to acknowledging the perception of worry. Peer‐support, team dynamics, and organisational culture also influence nurses' willingness to voice their concerns (Le Lagadec et al. 2024).

With technological advancements, increasing patient acuity, and a growing understanding of clinical deterioration, healthcare facilities have introduced algorithmic tools like PTTS to facilitate and standardise nursing care. We argue that such algorithmic tools may constrain nurses' higher‐order thinking skills, relegating nurse worry to the margins of clinical deterioration knowledge because it is often viewed as aesthetic knowledge rather than empirical and is thus undervalued (Smith et al. 2021). Aesthetic knowing is built on nursing experience, and this concept analysis has shown that clinical experience and organisational support is an important antecedent of nursing worry. According to Chua et al. (2023), years of experience are associated with greater accuracy in assessing patient conditions, reinforcing the idea that intuitive knowing, developed through past experiences, is a critical component of nursing practice. This aligns with the concept of the ‘clinical gaze,’ which involves using all of one's senses to thoroughly understand the person's medical condition in the current context, relying on the nurse's experience, and using higher‐order thinking skills (Langkjaer et al. 2021; Öhlén et al. 2021). By highlighting the role of nurse worry in recognising and responding to patient deterioration, we have provided important evidence‐based insights that can be used to inform future policy decisions and initiatives, a key element of concept analysis methodology (Hellman 2024).

In the current concept analysis, knowing the patient is a central antecedent to worry across various cultural and healthcare contexts. Developing a therapeutic relationship with patients enables nurses to gain a unique insight into the patient's specific needs, facilitating a more focused assessment. Ede et al. (2021) highlighted that knowing the person serves as a foundation for nurses to process information and make informed decisions regarding care escalation decisions.

Additionally, organisational support is pivotal in validating the concept of nurse worry in the current study. Nurses need to feel supported to escalate care when they are worried, especially if the patient's vital signs do not indicate an immediate concern. Researchers have emphasised the importance of a positive culture that encourages open communication and values nurses' assessments since this can empower them to act decisively when they detect deterioration (Chua et al. 2020; Newman et al. 2023a). Conversely, a poor culture instils fear and trepidation, leading to underreporting concerns with potentially detrimental delays in care escalation (Connell et al. 2016; Newman et al. 2023b).

Another important antecedent of worry is the need for adequate resources; these resources (which include time, staffing levels and physical resources) assist nurses in their ability to assess a patient and to make sound judgements. This is a particularly important point for low resources clinical settings, such as rural/remote areas (Australian Institute of Health and Wellbeing 2024). Commonly, such areas struggle with low staffing and high agency nurse usage (potentially impacting upon a nurse's ability to ‘know’ the patient) (Wakerman et al. 2019). Likewise, escalation processes may also be remote or via telehealth, adding complexity to the worry escalation processes (Scott et al. 2024). As such, careful consideration of the required antecedents identified in this article may assist low resourced areas to develop strong policy and processes around support, assessment, escalation, and validation of worry. We identified in this concept analysis the attributes of nurse worry as assessing, sensing, recognising, and processing. Assessment is both an antecedent and an attribute of worry, characterised by meaningful engagement with people as patients (Langkjaer et al. 2021; Odell et al. 2009). This encompasses objective clinical signs and subjective cues that may indicate deterioration, such as changes in behaviour or subtle shifts in communication (Currey et al. 2022). The ability to sense deterioration is described as an awareness or intuition that nurses develop over time, often informed by their sense of responsibility for the patient in their care (Dresser et al. 2023). Recognising clinical deterioration involves identifying signs and symptoms that deviate from expected care trajectories. This recognition is often accompanied by knowing something is amiss, which can prompt nurses to act (Liaw et al. 2011). Processing information related to patient care is a complex task that requires nurses to integrate both subjective and objective data, often leveraging peer support and organisational protocols to inform their decisions (Massey et al. 2016).

The consequences of nurse worry can be categorised into assured practice and proactive care, versus apprehensive practice and reactive care. Assured practice arises when nurses feel validated and are empowered to act on their concerns, leading to proactive interventions that can significantly improve patient outcomes (Jirwe et al. 2010). In contrast, apprehensive practice often results from a lack of support and validation, leading to hesitation and reactive care. Nurses may become anxious about voicing their concerns, fearing that their assessments will be undervalued or dismissed (Minyaev et al. 2021). This trepidation can result in delayed interventions, which may exacerbate deterioration and compromise safety. In providing a clear definition of nurse worry, we hope that organisational structures, policy developers, education providers, and future researchers not only see the role and value of the concept but also use our definition to improve the education of nurses and care of vulnerable patients.

Overall, a nurse's sense of worry is a sensitive indicator that a patient may be deteriorating and thus worry is essential for the safe delivery of care. A firm understanding of nurse worry and its complexities is important to ensure that health leaders and policymakers work towards improving the team and workplace culture, empowering nurses to act on their sense of worry.

## 6 Limitations

This concept analysis has a few limitations. Firstly, due to the nature of the concept analysis and its theoretical foundations, this analysis focuses exclusively on published works, some of which may not necessarily be empirical in nature. As such, the analysis of nurse worry was shaped by the expertise of the research team and their interpretation of the available literature. Secondly, our search identified only one primary study from a non‐Western setting (Chua et al. 2023), which may limit the generalisability of the findings to non‐Western or resource‐limited contexts. Nevertheless, the findings in Chua et al. (2023) suggest that nurses' experiences of worry in relation to clinical deterioration may share similarities across Western and non‐Western contexts. Future research should examine nurse worry in diverse cultural and healthcare settings to enhance the generalisability and applicability of the conceptual model. Lastly, this concept analysis examined nurse worry in the context of clinical deterioration. Nurses may exercise worry in many situations which this analysis may not capture. Nevertheless, by leveraging Rodgers' six phase model, the analysis applied a consistent approach to extraction and analysis, allowing for a conceptual model and descriptive statement to form.

## 7 Conclusion

Nurse worry is a complex process influenced by external and internal factors. A nurse's ability to exercise worry, and escalation of care based on worry is critical to safe clinical practice, especially in situations of clinical deterioration. This concept analysis identifies the surrogate terms, antecedents, attributes, and consequences of nurse worry, offering a conceptual framework for understanding the processes, influences, and barriers of this important clinical skill. A full descriptive statement of nurse worry is provided and can be summarised as ‘Worry captures a nurse's sense of knowing the patient, and is embodied via assessing, sensing, recognising, and processing of information, cues, and patterns’.

While this analysis provides valuable insights into nurse worry in clinical deterioration, the applicability of the findings may vary across different cultural and healthcare settings, including health services, policy, education, and academia. The context, culture, and respect for nursing care significantly impact how nurses worry. How worry is supported and acted on effectively can lead to either assured practice and proactive care or apprehensive practice and reactive care. Raising the profile of nurse worry and ensuring organisational support for it is essential in safe clinical care.

## Author Contributions

All authors have agreed on the final version and meet at least one of the following criteria: (1) substantial contributions to conception and design, acquisition of data, or analysis and interpretation of data; (2) drafting the article or revising it critically for important intellectual content.

## Ethics Statement

As a concept analysis, using published literature, no ethical clearance was required for this study.

## Consent

No patient participation within this study.

## Conflicts of Interest

The authors declare no conflicts of interest.

## Peer Review

The peer review history for this article is available at https://www.webofscience.com/api/gateway/wos/peer‐review/10.1111/jan.16956.

## Supporting information


Data S1.

## Data Availability

The data used for this research is available within the manuscript.

## Appendix A

Summary of articles and attributes, antecedents, and consequences of nurse worry.

**Table:**

Article #	Citation	Focus of article in relation to ‘worry’	Surrogate terms for ‘worry’ (Synonyms or interchanging terms)	Nurse worry Attributes (Features, characteristics)	Nurse worry Antecedents (Exists before or precedes)	Nurse worry Consequences (A result or effect of worried)
1	Al‐Moteri et al. (2019). Clinical deterioration of ward patients in the presence of antecedents: A systematic review and narrative synthesis.	To understand the antecedents of deterioration. Failure to recognise and respond to deterioration is of concern. Nurses do not always act on vital signs‐nurse worry may often be seen as a ‘more important’ indicator that leads to escalation.	Concern Clinical judgement Felt that something was wrong	Needing time to be able to make a decision Processing data	Decision making Building a believable case prior to escalation Fear of making the wrong decision	Escalation of care Failure to rescue
2	Chua et al. (2023). Automated rapid response system activation‐Impact on nurses' attitudes and perceptions towards recognising and responding to clinical deterioration: Mixed‐methods study.	There is also growing concern about the limitations of relying on vital signs alone to detect deteriorating patients. Nurses described often recognising early subtle signs of patient deterioration before abnormal changes which is facilitated by their knowledge about patients and intuition. This juxtaposes with medical officers reliance of objective indicators which can delay escalation of care.	Nurse worry Concern	Comprehensive assessment that goes beyond the numbers Knowing what is happening Early and subtle signs of deterioration	Nurses struggle to articulate intuition There is also the element of nurses worry being reflected as being inept for redundant activations Judgement	Escalation of care Failure to escalate care due to ambivalence or lack of judgement
3	Cioffi (2000a). Nurses' experiences of making decisions to call emergency assistance to their patients.	The experience of escalating care is explored, how nurses experience worry in relation to escalation.	Going Sour Something was wrong Gut feeling A sixth Sense Concern Anxious Nervous	Knowing the patient Using memories of previous patients with similar cues Pattern recognition	Skillful decision‐making Recognition of early signs of deterioration Complex decision‐making Detecting risks Fear of assessment being undervalued	Sitting on the patient Escalating via a Medical Emergency Team (MET) call
4	Cioffi (2000b). Recognition of patients who require emergency assistance: a descriptive study.	Exploration of what it means to be worried/seriously worried and perceptions of what leads to escalation of care based on this. Exploration of how nurses recognise and become worried.	Seriously worried Intuition Just know something in happening	Memories of patients with similar cues Experience Assessment Colour Agitation Clinical observations Asking the patient how they feel (feeling worse, impending doom)	Touching, feeling, sensing and knowing Analysis of cues in context of the person	Escalation of care via a MET call
5	Cioffi et al. (2010). ‘Changes of concern’ for detecting potential early clinical deterioration: a validation study.	Determining the content validity of ‘changes of concern’ used by nurses to call an emergency response.	Cause for concern Seriously worried Worrisome change Clinical status	Identification of: Noisy breathing Inability to talk in sentences Increased oxygen demands Agitation Impaired mentation Impaired cutaneous perfusion Not following expected trajectory New or escalating pain New symptom New observation	Recognition of change in the compensatory phase before any marked compromise. Non‐quantifiable evidence	Escalating care by ‘convincing a medical officer’
6	Cioffi et al. (2009). ‘Patients of concern’ to nurses in acute care settings: a descriptive study.	There is a desire to ‘validate’ the nurse worry criteria using objective clinical signs. Between 11% and 46% of Met calls are due to worry however it clinical usefulness is not well understood. (Article is pre‐early warning tool implementation).	Concern Does not look right Clinical judgement Felt something was wrong Seriously worried	Feeling uncertain – a cognitive state when an event cannot be adequately defined or categorised Noisy breathing, Inability to talk in sentences, Increase oxygen requirements Agitation Impaired mentation Impaired skin perfusion Not expected trajectory New or increasing pain New symptom or new observation	Assessment Validating ones feelings	Communicating uncertainty to medical officers may prevent escalation of care and impact on patient safety. Escalation of care Failure to rescue
7	Dalton et al. (2018). Factors that influence nurses' assessment of patient acuity and response to acute deterioration.	Nursing intuition was used to steer nurses accounts of the situation and assessments legitimised beliefs.	Nurse Intuition Knowing something is wrong Gut feeling	Knowing the patient Looking for cues Not as chatty as the day before Looking at the patient and knowing	Assessment Assessing worry against other signs such as early warning tools	Authenticate findings—nurses found it difficult to escalate when a secondary authentication could not be found Failure to escalate Escalate care
8	Douw et al. (2015). Nurses' worry or concern and early recognition of deteriorating patients on general wards in acute care hospitals: a systematic review.	The value of the worry criteria is inconsistent. There are barriers at play which prevent escalation of care, and worry alone is difficult to use as a trigger for escalation.	Intuitive knowing Does not look good Has a look in the eyes	Nurses know the patient. Ten indicates of worry include: Changes in breathing Changes in circulation Rigours Changes in mentation Agitation Pain No clinical progress Patient indicates feels unwell Subjective nurse observations	Assessment The nurse gaze Objective and subjective reasoning	Escalation of care through rapid response Better treatment outcomes
9	Douw et al. (2016). Nurses' ‘worry’ as predictor of deteriorating surgical ward patients: A prospective cohort study of the Dutch‐Early‐Nurse‐Worry‐Indicator‐Score. International.	The Dutch‐early‐nurse‐worry‐indicator prefaces that vital signs alone as inadequate for deterioration detection and escalation. Nurses must have avenues to exercise judgement.	Nurse worry Nurse concern Clinical judgement Clinical concern	Intuitive, unconscious and quick decision‐making Pattern recognition Leveraging past experiences	Assessment Foreseeing a risk	Escalation of care Failure to rescue and conversely unnecessary calls when judgement is not used
10	DeVita et al. (2017). Capturing early signs of deterioration: the Dutch‐early‐nurse‐worry‐indicator‐score and its value in the Rapid Response System.	Nurse worry is associated with unplanned intensive care admissions and is predictive of deterioration.	Presence of worry Nurse worry Feelings of concern	Following the steps for communication: Identifying Communicating – changes to communication Responding	Assessment Interprofessional communication Convincing a medical officer Increase vital sign frequency	Poor interprofessional collaboration Escalation of care/failure to rescue
11	Douw et al. (2018). Surgical ward nurses' responses to worry: An observational descriptive study.	The addition of ‘worry’ to the criteria allows for nurses to act upon intuition and exercise judgement.	Intuition Judgement without rationale Nurse concern Patient does not look good A look in the patients eyes	Anticipating deterioration Assessment Changes in breathing Change in circulation Rigours Change in mentation Deviation of vital signs Agitation Pain Unexpected trajectory Patient indicates not feeling well	Using experience Knowing without rationale Making skilled judgements Using cues	Judgement is created over time Escalation Communication
12	Dresser et al. (2023). Frontline Nurses' clinical judgement in recognising, understanding, and responding to patient deterioration: A qualitative study.	Worry is impacted by a nurses ability to notice, interpret, and respond to situations of patient deterioration.	Noticing Interpreting Responding	Assessment Frequent touch points with patient Interpreting the findings of assessment	Knowing the patient Using past experiences Leveraging the culture of team work Working to ones sense of responsibility	Activation of response Escalation of care Communicating with wider team
13	Ede et al. (2021). Human factors in escalating acute ward care: a qualitative evidence synthesis.	Human factors and escalation—worry as a ‘soft sign’ of deterioration.	Soft signs Awareness	Non‐numerical data Pale skin Respiratory patterns Blood loss Personality change Patient compliant Skin temperature Patient fatigue	Working from a baseline of knowledge about the patient The need for a strong culture, processes and medical response	Systemic issues such as staff workloads and care demands impede the detection of deterioration Poor team communication and functioning limited escalation of care Lack of consistent decision‐making impedes escalation of care
14	Jensen et al. (2022). Signs and symptoms, apart from vital signs, that trigger nurses' concerns about deteriorating conditions in hospitalised paediatric patients: A scoping review.	Worry as being a process outside of traditional vital signs.	Gut feeling Intuitive feeling A feeling that something is not right Gut instinct	Assessment A marriage of the clinical examination and intuition Intuition is not being able to describe the physical finding The patient is more sleepy Colour change Pain that worsens	Confidence to use their gut is built overtime Noticing behavioural cues Recognising risk	Escalation of care Influencing more junior nurses to exercise their clinical judgement
15	Langkjaer et al. (2021). Nurses' Experiences and Perceptions of two Early Warning Score systems to Identify Patient Deterioration—A Focus Group Study.	EWS systems need to be optimised and developed to support the complex care needs and patient safety, but there is a lack of knowledge about RNs' experiences and perceptions of these systems.	Concerned Gut feeling	Observations, being more concerns by what the data shows Experience Clinical gaze Using touch‐ feeling for a pulse Looking after paleness Listening to breathing	Meaningful Assessment	Modifying the score to justify escalation Escalation Communicating important knowledge
16	Minyaev et al. (2021). Could standing orders have a place? A phenomenological exploration of experienced ward‐based registered nurses' views on the escalation protocol for patient deterioration.	There is tension between the requirement of RNs to follow the escalation protocol and their desire to exercise clinical judgement in the management of specific situations, prior to involvement of the Medical Emergency Team (MET).	Gut feeling Intuitive awareness Concerned	Observations Decreasing blood pressure Context (postoperative) Monitoring over time	Assessment Making a judgement Decision making Following steps and fitting the criteria	Intervening early to avoid deterioration Worry is built over time Without worry, people could deteriorate Building knowledge
17	Odell et al. (2009). Nurses' role in detecting deterioration in ward patients: systematic literature review.	By describing what we know about current nursing observation practice through the process of a systematic literature review, we can better understand the context within which ward nurses' work and more fully appreciate the factors that may hinder or promote effective observation practice.	Intuitive knowing Seriously worried Something was wrong	Feeling ‘not right’ Colour Agitation Observations	Concerns about doing the ‘right thing’ Knowing the patient Past experiences Nursing credibility	Deterioration is avoided Escalation of care Advocacy
18	Peerboom et al. (2022). Surgical nurses' responses to worry: A qualitative focus group study in the Netherlands.	Worry is not well articulated or understood.	Something is not right Not normal	Assessment Observations (breathing, circulation, temperature, mentation, agitation, pain, unexpected trajectory) Patient indicates Nurse subjective	Sharing with another nurse Validation Confidence Supported by ‘check's’ Assessment	Escalation of care More frequent observations
19	Raymond et al. (2019). The meaning of ‘worried’ in MET call activations: A regional hospital examination of the clinical indicator.	Little is known about why nurses escalate a Medical Emergency Team (MET) response based on ‘worried’ criteria. This research has identified the exact clinical cause of ‘worried’ escalations, with evidence to support that clinical reasoning and patient deterioration are the main cause of ‘worried’ activation.	Worried Not quite right Intuition	Assessment Abnormal vital signs Clinical reasoning in relation to assessment	Abnormal vital signs Worry needs to be justified	Met call Care escalation
20	Romero‐Brufau et al. (2019). The fifth vital sign? Nurse worry predicts inpatient deterioration within 24 h.	The study aimed to evaluate whether the accuracy of nursing judgement, based on the ‘worried criterion’ in detecting impending physiological deterioration merits its inclusion in the electronic medical record.	Concern Nurse Judgement Sense of worry Intuition	Pattern recognition Analytical skills and intuition Change in the patients status	Assessment Years of experience were associated with greater accuracy around worry. The higher the worry factor the more care was escalated and the higher the rate of physician review	Escalation of care Transfer to ICU Validation of concerns and interventions
21	Schnock et al. (2021). Identifying nursing documentation patterns associated with patient deterioration and recovery from deterioration in critical and acute care settings.	Nurses frequently detect patient deterioration through intuitive perception influenced by familiarity with their patients and experiential understanding of the clinical course. Nurses' intuitive perceptions or observations of patients may not be formally communicated or documented. Study investigated the nature of these barriers by quantifying variations in nursing documentation patterns and confirming with nurses and physicians how those variations reflect clinical practice.	Concerned	Assessment Documentation: ‘If you're checking SpO2 and blood pressures more often, you're more worried about the patient. If you're commenting about the SpO2, that's bad.’	Validation of concerns	Escalation of care More, and more detailed documentation
22	Shiell et al. (2022). Exploration of a rapid response team model of care: A descriptive dual methods study.	To understand how the rapid response team was operationalised, and how members interacted with clinicians in inpatient hospital ward settings.	Staff concern Intuitive Clinician concern	Initial management Instigating interventions Administering fluids Repositioning	Intuition Confidence in a legitimate concern Making a decision to escalate	Escalation of care Comprehensive assessment Assessment and concern go hand in hand
